# Association of AISI and SIRI levels with mortality risk in patients with type 2 diabetes: A retrospective cohort study

**DOI:** 10.1097/MD.0000000000049713

**Published:** 2026-07-17

**Authors:** Sixian Wang, Mingjun Liu, Shaotao Chen, Qifan Guan, Yi Tan, Zhijie Li, Zhihao Dong, Ning Yang, Rongsheng Jiang, Yuxing Tai

**Affiliations:** a College of Acupuncture and Massage, Jiangxi University of Chinese Medicine, Nanchang, Jiangxi, China; b College of Acupuncture and Massage, Changchun University of Chinese Medicine, Changchun, Jilin, China.

**Keywords:** Aggregate Index of Systemic Inflammation (AISI), all-cause mortality, cardiovascular disease mortality, GBD, NHANES, Systemic Inflammation Response Index (SIRI), type 2 diabetes mellitus

## Abstract

Inflammation plays a key role in complications and organ damage in type 2 diabetes mellitus (T2DM). The Aggregate Index of Systemic Inflammation (AISI) and Systemic Inflammation Response Index (SIRI) have emerged as potential markers for inflammation and prognosis. This study aimed to assess the association between AISI, SIRI levels, and mortality in T2DM patients. This study combined a population-level time-trend analysis using Global Burden of Disease data from 1990 to 2021 with a retrospective cohort study using National Health and Nutrition Examination Survey data from 1999 to 2018 linked to the National Death Index mortality files. The Global Burden of Disease data revealed an upward trend in T2DM incidence, with a slight decline in mortality rates from 2003 onward. In the National Health and Nutrition Examination Survey cohort, higher AISI and SIRI levels correlated with increased risks of cardiovascular disease (CVD) and all-cause mortality. After adjusting for confounders, the highest quartile of both indices (Q4) showed significantly higher mortality risks: for AISI quartile 4 (Q4) vs Q1, hazard ratio (HR) = 1.46 for CVD and HR = 1.71 for all-cause mortality; for SIRI Q4 vs Q1, HR = 1.98 for CVD and HR = 2.13 for all-cause mortality. These findings suggest AISI and SIRI may serve as simple markers for risk stratification in T2DM.

## 1. Introduction

Modernization, urbanization, and rapid economic development have improved people’s quality of life, but they have also led to the widespread adoption of sedentary lifestyles, increased psychological stress and anxiety, and unhealthy dietary habits. These unhealthy lifestyles and dietary patterns are major drivers of the rapid increase in the incidence of type 2 diabetes mellitus (T2DM). According to the 2025 report of the International Diabetes Federation Diabetes Atlas, the prevalence of diabetes among adults aged 20 to 79 years worldwide was 12.2% in 2024, the majority of whom had T2DM. This figure is projected to rise to 17.1% by 2050.^[1]^ As a major public health issue, T2DM not only directly impacts population health but also significantly contributes to the onset and progression of various microvascular and macrovascular diseases, such as diabetic retinopathy, diabetic nephropathy, diabetic neuropathy, atherosclerosis, and cardiovascular disease (CVD).^[2]^ Approximately 32% of people with type 2 diabetes worldwide have CVD, including ischemic heart disease/coronary heart disease, heart failure, stroke, and peripheral artery disease.^[3]^ In the general population, the prevalence of CVD is approximately 7 to 8%, which is substantially lower than in people with T2DM.^[4]^ Mortality associated with these complications remains high. These findings underscore the need to better understand the inflammatory mechanisms underlying T2DM-related complications and to identify simple markers for risk stratification.

Hyperglycemia in T2DM patients induces glucotoxicity and increases the formation of advanced glycation end products, thereby promoting adipose tissue inflammation and systemic chronic low-grade inflammation, partly through reducing nitric oxide bioavailability and increasing the production of reactive oxygen species.^[5]^ Chronic immune-mediated inflammation, as one of the primary pathological features of T2DM, not only exacerbates the progression of diabetes and its associated complications through chronic low-grade inflammation but also increases the risk of cardiovascular events. Furthermore, the chronic hyperinflammatory state in T2DM patients contributes to the increased mortality associated with complications such as cancer and diabetic nephropathy.^[2]^ Although T2DM management typically relies on conventional metabolic markers such as fasting plasma glucose (FPG) and glycated hemoglobin (GHb [HbA1c]), clinical focus often centers on medication adjustments while overlooking the impact of changes in peripheral blood cell counts (particularly circulating immune cells) on patient prognosis. Currently, most patients rely on self-monitoring of blood glucose using fingerstick capillary glucose meters in primary care settings or at home. Only a minority undergo regular annual hospital-based health examinations, which predominantly assess biochemical parameters such as serum lipids, glucose, and uric acid. The prognostic value of peripheral blood cell counts in patients with T2DM has not received sufficient attention.

Given that this chronic inflammatory burden is reflected in peripheral blood cell counts, inflammation-related indices derived from routine complete blood counts (CBCs) may provide additional prognostic information in T2DM. The Aggregate Index of Systemic Inflammation (AISI: neutrophils [NEU] × platelets [PLT] × monocytes [MONO]/lymphocytes [LYM]) and the Systemic Inflammation Response Index (SIRI: NEU × MONO/LYM) are 2 blood cell count-derived composite indices that are increasingly used to assess systemic inflammatory status from routine CBCs. These indices are not only readily accessible but also dynamically reflect the course of the inflammatory process. AISI, as a novel prognostic biomarker, has emerged as a novel blood cell count-derived index of systemic inflammation and has been investigated in diseases such as idiopathic pulmonary fibrosis (IPF). Research indicates that AISI has been reported to discriminate between patients with IPF and healthy individuals, and its levels are independently associated with the presence of IPF and with impaired lung function, a marker of disease severity.^[6,7]^ Furthermore, AISI has been reported as a reliable predictor of mortality in patients with viral pneumonia, such as coronavirus disease 2019-related pneumonia.^[8]^ SIRI, as a novel composite inflammatory index, has demonstrated promising predictive capabilities in studies of chronic infections, stroke, cancer, and CVD, and elevated SIRI levels have also been associated with increased all-cause and cardiovascular mortality in large community-based cohorts.^[9–11]^ Research also indicates that SIRI can serve as a screening marker for coronary heart disease in symptomatic young adults.^[12]^ Taken together, these CBC-derived indices offer simple, inexpensive tools to capture systemic inflammation and may be useful for risk stratification in T2DM.

However, current research on the application of AISI and SIRI in patients with T2DM remains limited, particularly regarding the extent to which they independently predict all-cause and cardiovascular mortality in this population and their ability to discriminate mortality risk. Therefore, this study aims to investigate whether AISI and SIRI are independently associated with all-cause and cardiovascular mortality in patients with T2DM and to compare their predictive performance for these mortality outcomes. Through this research, we hope to identify simple and effective inflammation-related indices for clinical practice, with the goal of improving prognostic assessment and guiding intervention strategies in patients with T2DM.

## 2. Methods

### 2.1. Study design and data sources

This study comprised 2 components: a population-level trend analysis based on Global Burden of Disease (GBD) 2021 data, and a retrospective cohort analysis using National Health and Nutrition Examination Survey (NHANES) 1999 to 2018 data linked to the National Death Index (NDI) mortality files. The GBD data were used to examine temporal trends in the incidence and mortality of T2DM in the United States (U.S.) from 1990 to 2021. The NHANES cohort was employed to investigate the associations of baseline AISI and SIRI levels with all-cause and cardiovascular mortality among adults with T2DM.

### 2.2. Study population

The GBD database uses systematic modeling to estimate the incidence, prevalence, mortality, and disability burden of major diseases and risk factors at national and global levels. In this study, GBD 2021 data from 1990 to 2021 were used to assess population-level trends in T2DM incidence and mortality in the U.S..

NHANES is a nationally representative, probability-based survey conducted by the National Center for Health Statistics (NCHS) in 2-year cycles among noninstitutionalized civilians in the U.S.. In this study, data from 10 consecutive NHANES cycles between 1999 and 2018 were linked to NDI mortality files for the retrospective cohort analysis.

**Figure 1.**
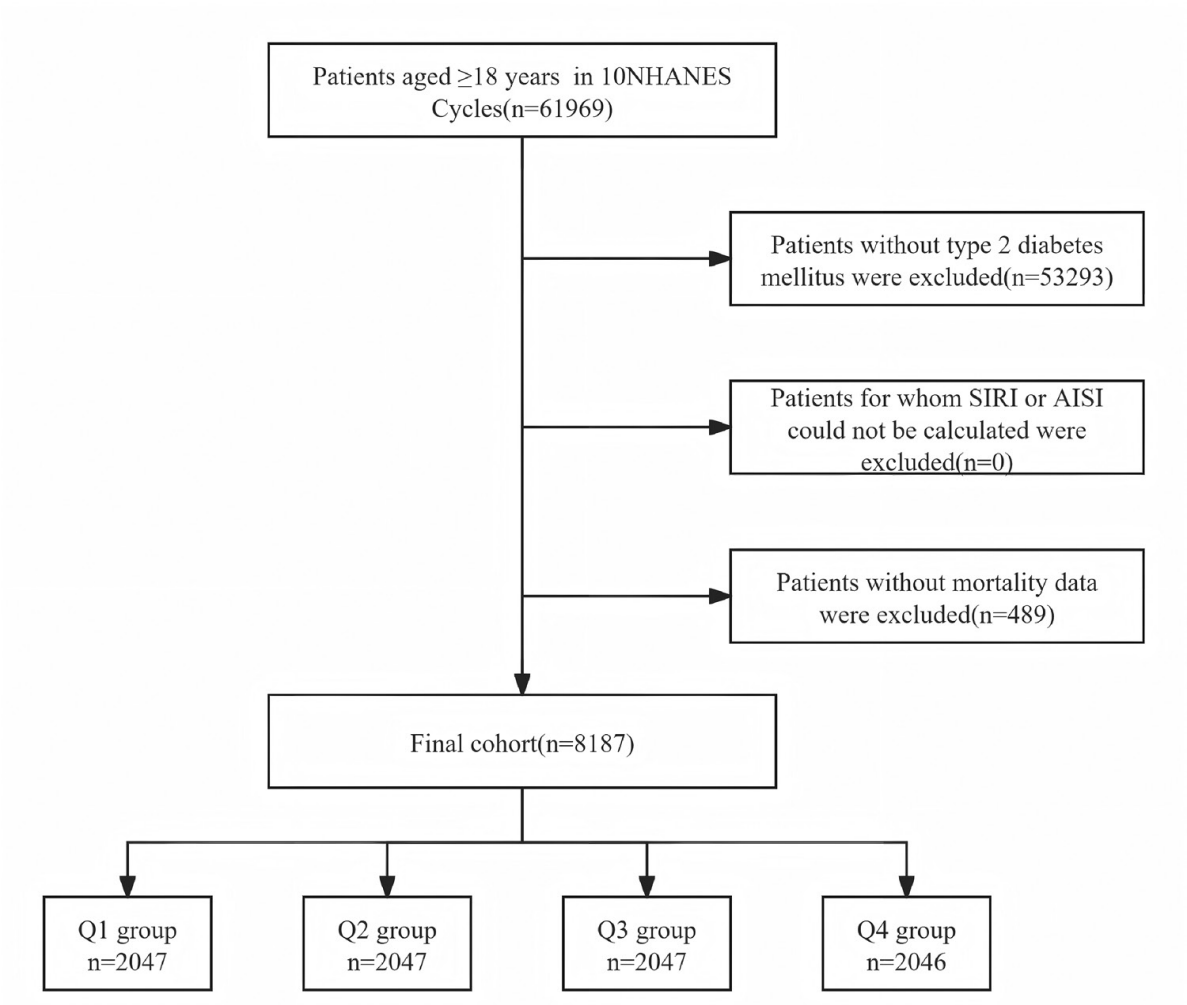
Flowchart of study population selection from the NHANES 1999 to 2018. AISI = Aggregate Index of Systemic Inflammation, n = number of participants, NHANES = National Health and Nutrition Examination Survey, SIRI = Systemic Inflammation Response Index.

From 1999 to 2018, NHANES enrolled 61,969 participants aged 18 years and older. A total of 53,293 participants without T2DM were excluded. T2DM was defined according to the American Diabetes Association criteria as any of the following^[13]^: GHb ≥ 6.5%, FPG ≥ 7.0 mmol/L (126 mg/dL), current use of insulin or other glucose-lowering medication, or a self-reported physician diagnosis of diabetes. Participants with missing NEU, LYM, MONO, or PLT counts were further excluded; however, no participants met this exclusion criterion (n = 0). In addition, 489 participants with missing mortality data were excluded. Ultimately, 8187 participants with T2DM were included in the NHANES-based retrospective cohort analysis (Fig. 1).

### 2.3. Study variables

Participants self-reported age, sex, race, marital status, education level, poverty-to-income ratio, smoking status, alcohol consumption, and antihypertensive medication use. Age was categorized as 18 to 44, 45 to 64, 65 to 74, and ≥ 75 years. Body mass index (BMI) was categorized as underweight, normal weight, overweight, or obese, and abdominal obesity was defined according to waist circumference. Laboratory metrics, including GHb, FPG, NEU count, LYM count, MONO count, and PLT count, were measured using automated blood analyzers. AISI was calculated as NEU × PLT × MONO/LYM, and SIRI was calculated as NEU × MONO/LYM. Participants were categorized into quartiles according to baseline AISI and SIRI levels. Detailed procedures for obtaining laboratory measurements are available on the NCHS website. Comorbidities and medication use were determined based on participants’ self-reported physician diagnoses within the past 12 months.

Participant mortality information was obtained from the NHANES publicly linked mortality file provided by the U.S. Centers for Disease Control and Prevention NDI. Follow-up time was calculated from baseline examination to death or censoring through December 31, 2019. Participants who were alive at the end of follow-up were censored. Causes of death were coded according to the International Classification of Diseases, Tenth Revision. All-cause mortality encompassed any underlying cause of death, while CVD mortality was explicitly categorized using International Classification of Diseases, Tenth Revision codes I00 to I09, I11, I13, I20–I51, and I60 to I69. Participants with missing NEU, LYM, MONO, or PLT counts were excluded; however, no participants met this exclusion criterion. Participants with missing mortality information were also excluded. For covariates with missing values, multiple imputation was performed to reduce potential bias due to missing data.

### 2.4. Statistical analysis

All NHANES analyses accounted for the complex survey design, including sampling weights, strata, and primary sampling units, according to the recommendations of the NCHS. Continuous variables are presented as mean ± standard deviation, and categorical variables are presented as counts and percentages. Baseline characteristics across AISI and SIRI quartiles were compared using analysis of variance for continuous variables and chi-square (*χ*^2^) tests for categorical variables.

Kaplan–Meier survival curves and Cox proportional hazards models were used to evaluate the associations of AISI and SIRI with all-cause and cardiovascular mortality. Three Cox models were constructed: Model 1, unadjusted; Model 2, adjusted for age and sex; and Model 3, additionally adjusted for ethnicity, education level, poverty status, BMI, abdominal obesity, smoking status, alcohol consumption, and dyslipidemia. Hazard ratios (HRs) and 95% confidence intervals (CIs) were reported. The proportional hazards assumption was assessed using Schoenfeld residuals, and multicollinearity was evaluated using variance inflation factors. Subgroup analyses were performed according to age, sex, obesity status, smoking status, alcohol consumption, and dyslipidemia. Age was stratified as 18 to 44, 45 to 64, 65 to 74, and ≥ 75 years. Obesity, smoking status, alcohol consumption, and dyslipidemia were categorized according to the definitions used in the baseline characteristics. Potential effect modification was assessed by adding interaction terms between AISI or SIRI quartiles and subgroup variables to the Cox models.

Because this was a secondary analysis of publicly available NHANES data, no a priori sample size calculation was performed. Instead, all eligible adults with T2DM from NHANES 1999 to 2018 who met the inclusion criteria were included. During follow-up, 2465 all-cause deaths and 706 cardiovascular deaths occurred. The number of outcome events was considered sufficient for the multivariable Cox models based on the events-per-variable principle.

In the GBD section, we used age-standardized incidence rates and age-standardized mortality rates as primary outcome measures, and employed Joinpoint regression models to estimate annual percentage changes (APC) for the period 1990 to 2021.

All NHANES analyses were performed using R software (version 4.4.3; R Foundation for Statistical Computing). GBD data were analyzed using the Joinpoint Regression Program (version 5.4.0; National Cancer Institute). A 2-sided *P* < .05 was considered statistically significant.

### 2.5. Ethics statement

NHANES protocols were approved by the NCHS Research Ethics Review Board, and all participants provided written informed consent. The present study used publicly available de-identified NHANES data and aggregated GBD data; therefore, no additional institutional review board approval was required.

## 3. Results

### 3.1. Time trends in the incidence and mortality of T2DM

**Figure 2.**
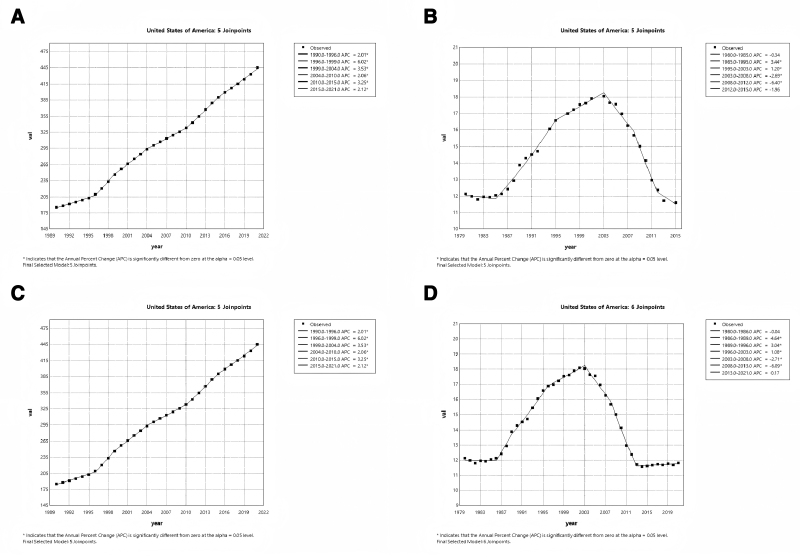
Temporal trends in T2DM incidence and mortality in the U.S. from 1990 to 2021 based on GBD 2021 data. (A) ASIR of T2DM per 100,000 population. (B) ASMR of T2DM per 100,000 population. APC values indicate the rate of change for each segment. (C) Number of new T2DM cases in the U.S. population over time, with APC values for each trend segment. (D) ASMR among U.S. adults aged 65 years and older with T2DM, showing a reversal of the declining trend since 2015 (APC = 0.17). APC = annual percentage change, ASIR = age-standardized incidence rate, ASMR = age-standardized mortality rate, GBD = Global Burden of Disease, T2DM = type 2 diabetes mellitus, U.S. = United States.

From 1990 to 2021, the age-standardized incidence rate of T2DM continued to rise across the entire U.S. population. Compared to the rapid growth observed between 1996 and 1999, the rate of increase slowed after 1999 due to factors including advances in medical technology, lifestyle changes, and increased public health interventions (APC = 6.02 for 1996–1999; APC = 2.12 for 2015–2021). The APC remained positive, indicating that the burden of new T2DM cases in the U.S. population continues to increase (Figs. 2A and 2C).

From 1990 to 2021, the age-standardized mortality rate for T2DM in the U.S. population showed a declining trend starting in 2003 (APC = −2.69). However, since 2012, the rate of decline has gradually slowed (APC = −1.96) (see Fig. 2B). This deceleration was particularly pronounced among individuals aged 65 and older. Since 2015, mortality rates among U.S. adults aged 65 and older with T2DM have even begun to rise (APC = 0.17) (see Fig. 2D).

### 3.2. Baseline characteristics

Among the 8187 patients with T2DM, the age distribution across the 4 SIRI quartiles (Q1: n = 2047; Q2: n = 2047; Q3: n = 2047; Q4: n = 2046) showed significant differences (*P* < .001). The overall population was predominantly middle-aged (45–64 years, 42.2%), followed by older adults aged 65 to 74 years (26.0%), adults aged 18 to 44 years (13.0%), and those aged ≥ 75 years (18.9%). The proportion of ≥ 75-year-olds progressively increased from 11.0% in Q1 to 29.0% in Q4 with rising SIRI levels, indicating an older age structure in higher SIRI groups. Regarding sex distribution, females accounted for 48.3% and males for 51.7%. The proportion of females decreased from 57.4% in Q1 to 38.6% in Q4 as SIRI levels increased. Racial composition was dominated by non-Hispanic Whites (35.8%) and non-Hispanic Blacks (25.1%). Non-Hispanic Whites constituted the highest proportion in the Q4 group (52.6%), while the proportion of non-Hispanic Blacks decreased with increasing SIRI (Q1: 41.3% vs Q4: 15.3%). Within AISI quartiles, age distribution trends mirrored SIRI: the proportion aged ≥ 75 increased from 13.9% in Q1 to 23.6% in Q4, while the 18 to 44 age group slightly rose to 14.2% in Q4. Gender distribution showed no significant differences across AISI groups (*P* = .184), with the female proportion remaining stable at approximately 48%. Racial distribution similarly exhibited an increasing trend in non-Hispanic White individuals within AISI groups, reaching 48.7% in Q4. Conversely, the proportion of non-Hispanic Black individuals decreased from 37.5% in Q1 to 17.0% in Q4. Additionally, no significant differences were observed in marital status, education level, or poverty status across the 2 index groups. However, both SIRI and AISI levels increased with higher BMI and a greater prevalence of abdominal obesity. Smoking and alcohol consumption also contributed to elevated inflammatory states, suggesting higher metabolic risks among patients with high inflammation. Further baseline characteristics of the study population are detailed in Tables S1 and S2, Supplemental Digital Content 3.

### 3.3. Inflammatory markers and mortality

**Figure 3.**
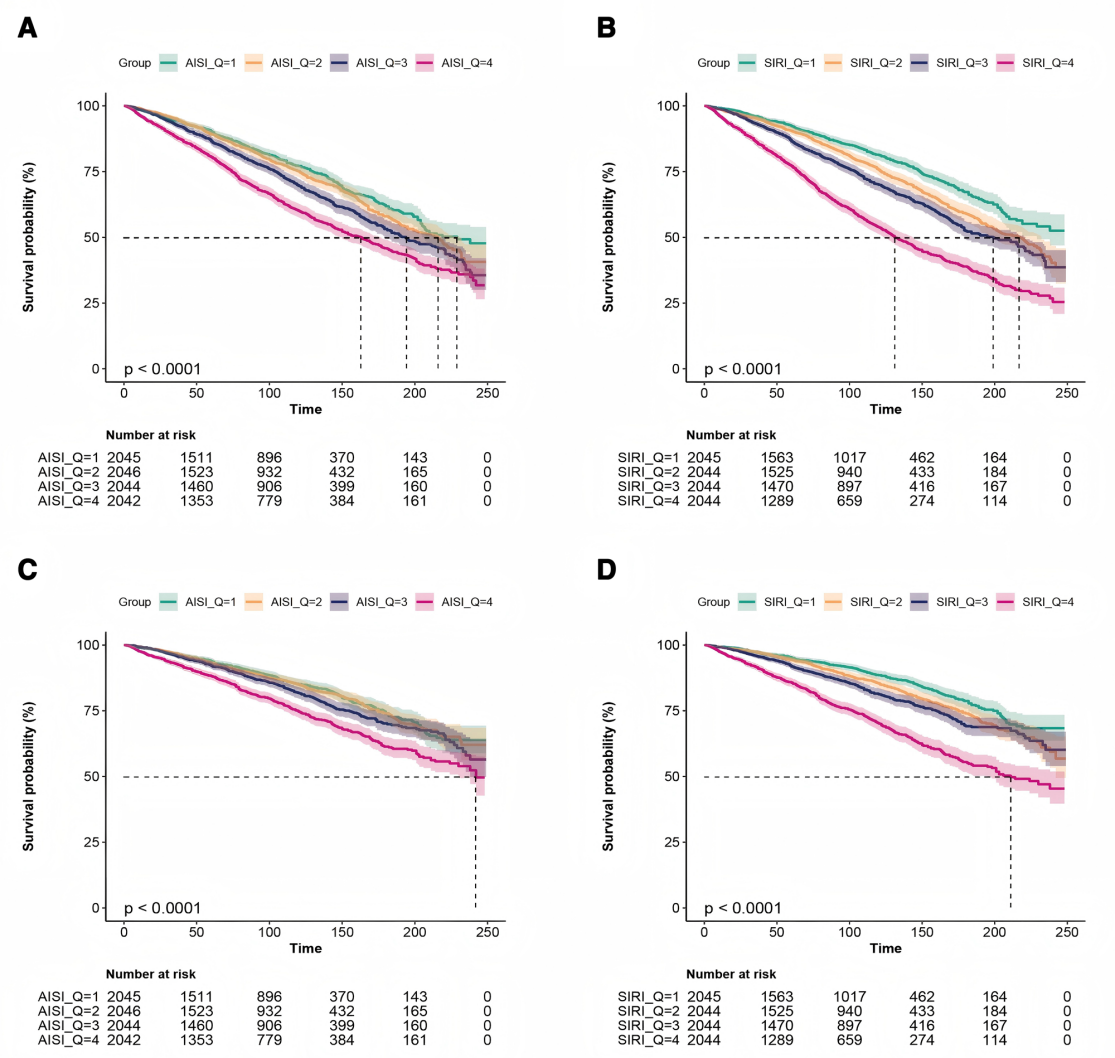
Kaplan–Meier survival curves for all-cause and CVD mortality according to quartiles of the AISI and SIRI in adults with T2DM, NHANES 1999 to 2018. (A) All-cause mortality by AISI quartiles (Q1–Q4). (B) All-cause mortality by SIRI quartiles (Q1–Q4). (C) CVD mortality by AISI quartiles (Q1–Q4). (D) CVD mortality by SIRI quartiles (Q1–Q4). AISI was calculated as (NEU × MONO × PLT)/LYM. SIRI was calculated as (NEU × MONO)/LYM. Differences between groups were assessed using the log-rank test. AISI = Aggregate Index of Systemic Inflammation, CVD = cardiovascular disease, LYM = lymphocytes, MONO = monocytes, NEU = neutrophils, NHANES = National Health and Nutrition Examination Survey, PLT = platelets, SIRI = Systemic Inflammation Response Index, T2DM = type 2 diabetes mellitus.

Kaplan–Meier survival curves (Fig. 3) showed progressively lower survival probabilities among participants with higher AISI and SIRI quartiles, with significant differences across groups for both all-cause and cardiovascular mortality.

For all-cause mortality, higher AISI levels were associated with increased mortality risk (Table S3, Supplemental Digital Content 4). In the unadjusted model, compared with Group 1, the HRs were 1.13 (95% CI: 1.00–1.27; *P* = .058), 1.32 (95% CI: 1.17–1.49; *P* < .001), and 1.87 (95% CI: 1.67–2.09; *P* < .001) for Groups 2, 3, and 4, respectively. After full adjustment in Model 3, the association remained significant for Group 4 (HR = 1.71, 95% CI: 1.47–2.00; *P* < .001), whereas the associations for Group 2 (HR = 1.08, 95% CI: 0.91–1.27; *P* = .376) and Group 3 (HR = 1.17, 95% CI: 0.99–1.37; *P* = .060) were not statistically significant.

SIRI showed a stronger graded association with all-cause mortality (Table S4, Supplemental Digital Content 5). In the unadjusted model, compared with Group 1, the HRs were 1.33 (95% CI: 1.17–1.51; *P* < .001), 1.62 (95% CI: 1.43–1.84; *P* < .001), and 2.86 (95% CI: 2.55–3.22; *P* < .001) for Groups 2, 3, and 4, respectively. After full adjustment in Model 3, all higher SIRI groups remained significantly associated with increased all-cause mortality risk: Group 2, HR = 1.26 (95% CI: 1.06–1.49; *P* = .008); Group 3, HR = 1.39 (95% CI: 1.17–1.64; *P* < .001); and Group 4, HR = 2.13 (95% CI: 1.81–2.51; *P* < .001).

For cardiovascular mortality, higher AISI levels were also associated with increased risk (Table S5, Supplemental Digital Content 6). In Model 3, only Group 4 remained significantly associated with cardiovascular mortality compared with Group 1 (HR = 1.46, 95% CI: 1.10–1.95; *P* = .009), whereas Group 2 (HR = 0.93, 95% CI: 0.69–1.26; *P* = .633) and Group 3 (HR = 1.00, 95% CI: 0.74–1.35; *P* = .995) were not significantly associated with cardiovascular mortality.

Similarly, higher SIRI levels were associated with increased cardiovascular mortality risk (Table S6, Supplemental Digital Content 7). After full adjustment in Model 3, Group 4 had a significantly higher risk of cardiovascular mortality than Group 1 (HR = 1.98, 95% CI: 1.46–2.69; *P* < .001). The associations for Group 2 (HR = 1.16, 95% CI: 0.84–1.59; *P* = .371) and Group 3 (HR = 1.18, 95% CI: 0.86–1.62; *P* = .311) were not statistically significant.

Taken together, both AISI and SIRI were associated with increased all-cause and cardiovascular mortality among adults with T2DM. The highest quartile of both indices remained significantly associated with mortality outcomes after multivariable adjustment. For all-cause mortality, SIRI showed a clearer dose-response pattern, whereas for cardiovascular mortality, the excess risk was mainly observed in the highest quartile of AISI and SIRI.

Model diagnostics were performed for the fully adjusted Cox models. For all-cause mortality, the proportional hazards assumption was not significantly violated for either SIRI or AISI based on Schoenfeld residuals. For cardiovascular mortality, the global Schoenfeld tests were not significant, although the main exposure terms for SIRI and AISI showed evidence of potential non-proportionality. Therefore, the HRs for cardiovascular mortality should be interpreted as average associations over the follow-up period. Multicollinearity was not evident in the fully adjusted models, as all maximum adjusted generalized variance inflation factor values were below 2 (Table S7, Supplemental Digital Content 8).

### 3.4. Subgroup analysis

To further examine the associations of SIRI and AISI with all-cause and cardiovascular mortality, participants were stratified by age, sex, obesity, smoking and drinking habits, and the presence of dyslipidemia. The associations between these inflammatory markers and both all-cause and cardiovascular mortality were generally consistent across these strata.

Both SIRI and AISI were positively correlated with all-cause mortality and cardiovascular mortality. Consistent trends were observed across subgroups for both indices, although AISI exerted a more pronounced effect on participants with obesity and those who consumed alcohol, particularly in the alcohol-consuming group where its impact was especially significant. In contrast, SIRI’s influence was relatively balanced across all subgroups (Fig. S1–S4, Supplemental Digital Content 1).

## 4. Discussion

Although T2DM-related mortality has declined since 2003, the rate of decrease has slowed in recent years, and mortality has begun to rebound, particularly among individuals aged ≥ 65 years.GBD findings further indicate that despite advances in T2DM treatment, the overall disease burden and mortality risk among patients with T2DM remain substantial. Early identification of high-risk individuals and refinement of risk stratification tools remain critical clinical priorities. Therefore, we evaluated AISI and SIRI as blood cell count-derived inflammatory indices for assessing long-term mortality risk in patients with T2DM. Our results demonstrate that elevated AISI and SIRI levels in patients with T2DM are associated with increased all-cause and cardiovascular mortality, and these associations persist after full adjustment for confounding factors. These associations remained consistent across subgroups defined by age, sex, and obesity. Taken together with the long-term trend of increasing T2DM incidence and a slowing decline in mortality identified in the GBD analysis, our NHANES findings further show at the individual level that blood cell-derived indices of chronic inflammatory status have substantial predictive value for adverse outcomes in the growing T2DM population.

At present, the concept that patients with T2DM exist in a state of chronic low-grade inflammation has been widely accepted; therefore, the impact of inflammation on adverse prognosis in T2DM has also received extensive attention. Previous studies have shown that the neutrophil percentage-to-albumin ratio is significantly associated with all-cause mortality, whereas its association with CVD mortality does not reach statistical significance.^[14]^ Recent studies have found that AISI can be used to assess the risk of cardiovascular events and incident CVD in patients with chronic conditions. Evidence suggests that AISI can serve as an an independent predictor of prognosis in patients with CVD and is significantly associated with cardiovascular mortality in individuals with hypertension.^[15,16]^ As an inflammatory index, AISI incorporates PLT counts into its calculation. In T2DM, PLT hyperreactivity and MONO-driven inflammatory responses represent key mechanisms contributing to fatal atherothrombotic cardiovascular events.^[17–19]^ Therefore, AISI, as an inflammatory index, may reflect PLT activation and innate immune activation in patients with T2DM, thereby indicating the extent of vascular endothelial injury and thrombosis formation. In contrast, SIRI places greater emphasis on myeloid cell–mediated inflammatory responses and has also been shown to predict all-cause and cardiovascular mortality.^[20]^

The chronic inflammatory state of T2DM is mainly characterized by altered MONO function, elevated levels of pro-inflammatory cytokines, and enhanced PLT activation.^[21]^ These pro-inflammatory and prothrombotic alterations are major contributors to the development and progression of T2DM-related complications, particularly diabetic nephropathy and cardiovascular lesions.^[22]^ In patients with T2DM, persistent hyperglycemia continuously stimulates NEU and MONOs, leading to the production of large amounts of pro-inflammatory cytokines. Meanwhile, activated NEU and MONO can further drive T and B LYM to produce cytokines, including tumor necrosis factor-alpha and interleukin-6, thereby maintaining a state of chronic inflammation and increasing susceptibility to infection.^[23]^ Elastase and cytokines released by NEU and MONO can accelerate the progression of CVD,^[24–26]^ while also interfering with insulin signaling pathways and aggravating insulin resistance and metabolic dysregulation.^[27–29]^ In recent years, with increasing research into inflammation, studies have shown that neutrophil extracellular traps released by activated NEU can also promote oxidative stress, inflammation, and thrombosis, ultimately leading to endothelial injury.^[30,31]^ Under hyperglycemic conditions, MONO from patients with T2DM exhibit impaired chemotaxis and transendothelial migration, and defective reverse transendothelial migration may be related to integrin activation and increased junctional adhesion molecule-3 expression.^[32]^ In parallel, chronic hyperglycemia can promote T-cell activation and infiltration into target tissues, inducing the release of pro-inflammatory cytokines such as interleukin-17A, thereby aggravating renal and vascular dysfunction.^[33]^ In addition, oxidative stress induced by hyperglycemia and insulin resistance can promote PLT dysfunction and hyperreactivity.^[34,35]^ Together, these abnormalities contribute to vascular endothelial injury and the progression of atherosclerosis.^[36]^

Our research indicates that both AISI and SIRI reflect systemic inflammatory burden in patients with T2DM and are associated with all-cause and cardiovascular mortality. In clinical practice, these indices can be calculated from a simple CBC, allowing identification of patients with elevated systemic inflammation and assisting clinicians in risk assessment and timely intervention. Against the backdrop of persistently rising T2DM incidence and slowing declines in T2DM-related mortality, risk stratification using widely available, low-cost inflammatory indices such as AISI and SIRI holds promise for improving the identification of high-risk patients at the population level and ultimately enhancing prognosis and quality of life. T2DM is characterized by chronic low-grade inflammation, which differs from acute inflammation driven by localized infection or tissue injury. While antibiotic therapy is appropriate for localized infection-driven inflammation, long-term antibiotic use is not an appropriate strategy for attenuating chronic inflammation in T2DM. By contrast, when selecting glucose-lowering agents, drugs with additional anti-inflammatory properties may be considered. Metformin, one of the most commonly used first-line glucose-lowering agents, exerts well-documented anti-inflammatory effects.^[37]^ Its anti-inflammatory actions are thought to involve activation of adenosine monophosphate-activated protein kinase with subsequent inhibition of downstream pro-inflammatory signaling pathways, including mechanistic target of rapamycin and nuclear factor kappa B. In addition, several studies suggest that metformin also exerts anti-inflammatory effects that are at least partly independent of adenosine monophosphate-activated protein kinase.^[38]^ Furthermore, recent studies indicate that glucagon-like peptide-1 receptor agonists not only lower blood glucose by enhancing insulin secretion and reducing appetite but also exert anti-inflammatory effects and provide cardiovascular and renal protection. Emerging evidence suggests that they may also confer potential benefits in liver disease, obstructive sleep apnea, chronic respiratory diseases, neurodegenerative and psychiatric disorders, reproductive dysfunction, obesity-related cancers, and sepsis.^[39,40]^ Sodium-glucose cotransporter-2 inhibitors have been shown to exert cardiovascular protective effects, at least in part, by attenuating low-grade chronic inflammation.^[41,42]^ Our study extends the application of these indices to cardiovascular outcomes in patients with T2DM, providing a basis for future research that should be validated in multicenter prospective cohorts and randomized controlled trials.

AISI and SIRI are derived from routine CBC parameters and are characterized by easy accessibility, low cost, and strong clinical applicability, making them convenient tools for risk stratification in patients with T2DM. By combining population-level trend analyses from the GBD database with individual-level cohort analyses from the NHANES database, the present study further validated the associations of AISI and SIRI with mortality risk in patients with T2DM against the background of a continuously increasing T2DM disease burden and a slowing decline in T2DM-related mortality, thereby providing more comprehensive epidemiological evidence for the use of AISI and SIRI in mortality risk assessment among patients with T2DM. The primary data source of this study was the NHANES database, which includes relatively complete demographic information, laboratory measurements, and mortality follow-up data. As a nationally representative survey, NHANES adopts standardized data collection procedures and laboratory testing methods, which helps improve the reliability of the study findings and outcome assessment. In addition, the complex sampling design of NHANES was fully considered in the statistical analyses, and multivariable Cox regression models, subgroup analyses, proportional hazards assumption testing, and multicollinearity assessment were performed to further enhance the robustness of the results.

Our study also has several limitations. First, as a retrospective analysis based on NHANES data, residual confounding cannot be completely excluded despite adjustment using multivariable Cox regression models. We plan to further evaluate these associations in prospective cohort studies and, where feasible, randomized controlled trials. Second, a single baseline blood sample may not fully capture longitudinal changes in inflammatory status, as substantial variation in blood cell indices may occur during long-term follow-up.

## 5. Conclusion

The combined use of AISI and SIRI may provide a more objective and comprehensive assessment of systemic inflammation in patients with T2DM. In adult patients with T2DM, elevated AISI and SIRI levels were significantly associated with increased risks of cardiovascular and all-cause mortality. Based on our findings, AISI and SIRI values may serve as simple early warning markers of poor prognosis in patients with T2DM. These findings highlight the potential clinical value of targeting chronic low-grade inflammation and hyperglycemia-induced target organ damage when developing strategies to prevent T2DM-related complications. However, these results require further validation in prospective cohort studies and randomized clinical trials.

## Author contributions

**Conceptualization:** Sixian Wang, Mingjun Liu, Yuxing Tai.

**Data curation:** Yi Tan, Zhihao Dong, Ning Yang.

**Methodology:** Mingjun Liu.

**Supervision:** Mingjun Liu.

**Software:** Shaotao Chen, Qifan Guan.

**Visualization:** Shaotao Chen, Qifan Guan.

**Writing – original draft:** Sixian Wang, Yuxing Tai.

**Writing – review & editing:** Zhijie Li, Rongsheng Jiang.






















