# Supplementary figures and images for: Association of AISI and SIRI levels with mortality risk in patients with type 2 diabetes: A retrospective cohort study
Source: Medicine (Baltimore). 2026 Jul 17;105(29):e49713. doi: 10.1097/MD.0000000000049713 (PMC13384559; doi:10.1097/MD.0000000000049713)

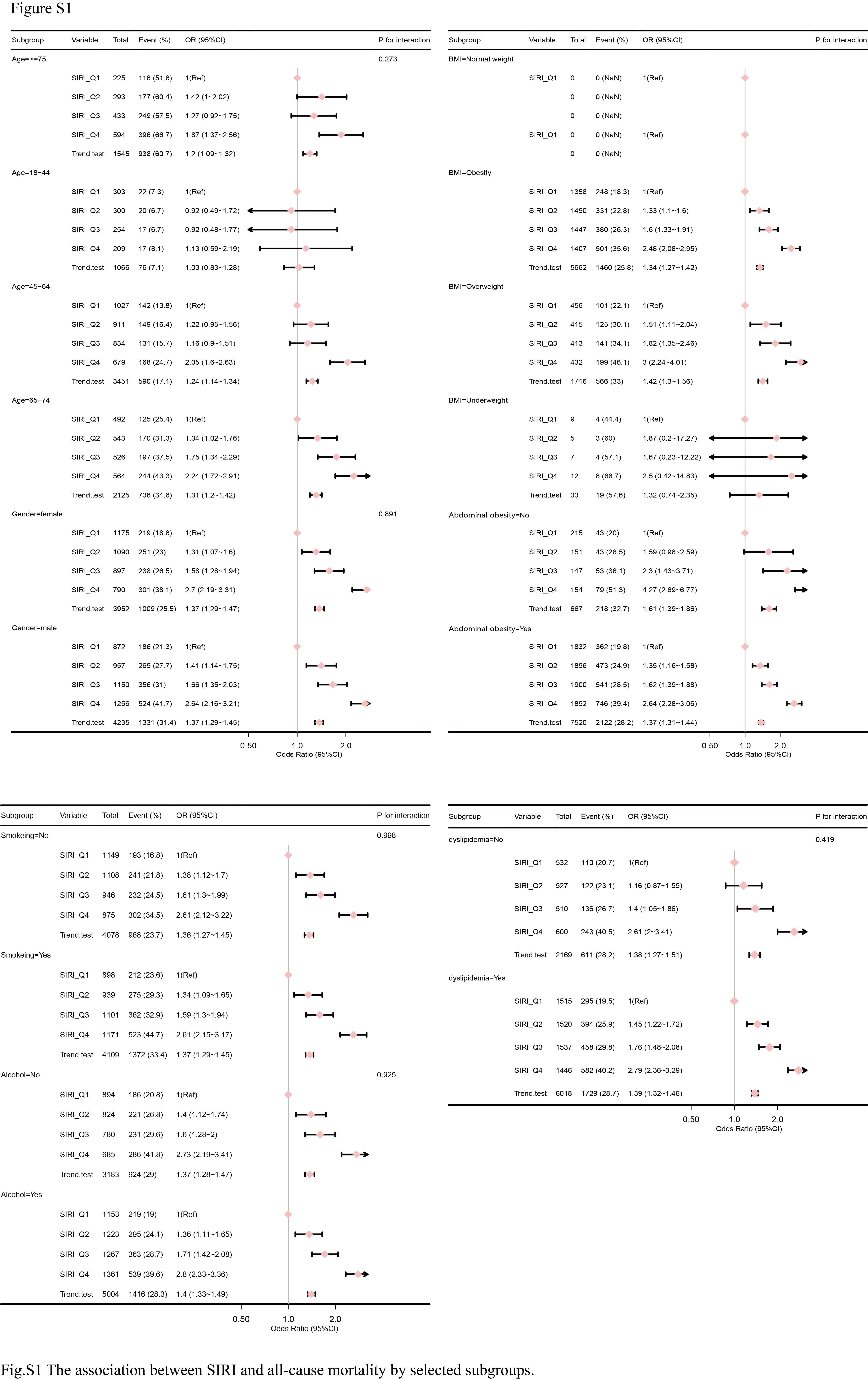

Supplement: Supplementary file 1 [file medi-105-e49713-s001.tiff]

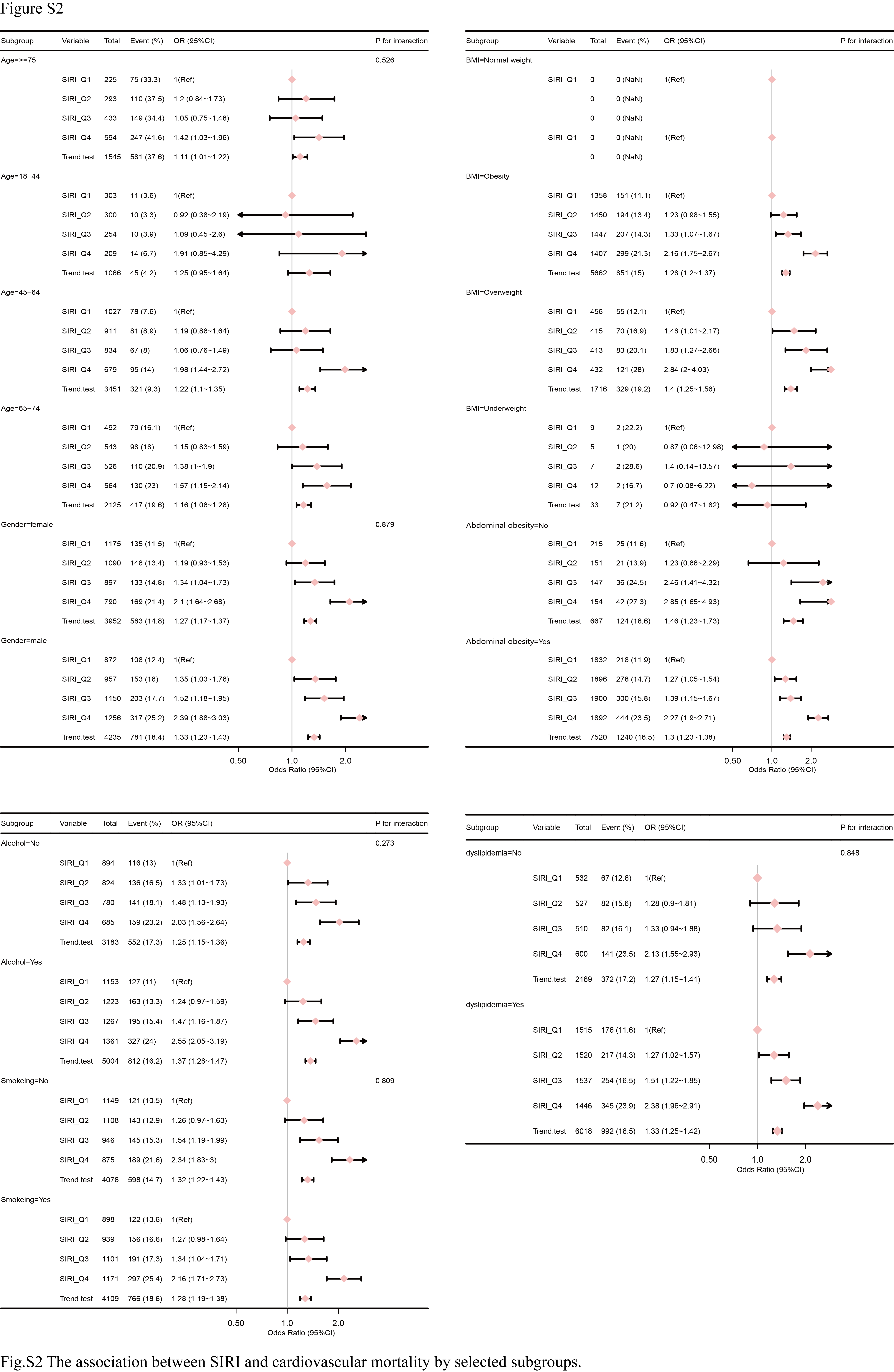

Supplement: Supplementary file 9 [file medi-105-e49713-s009.tiff]

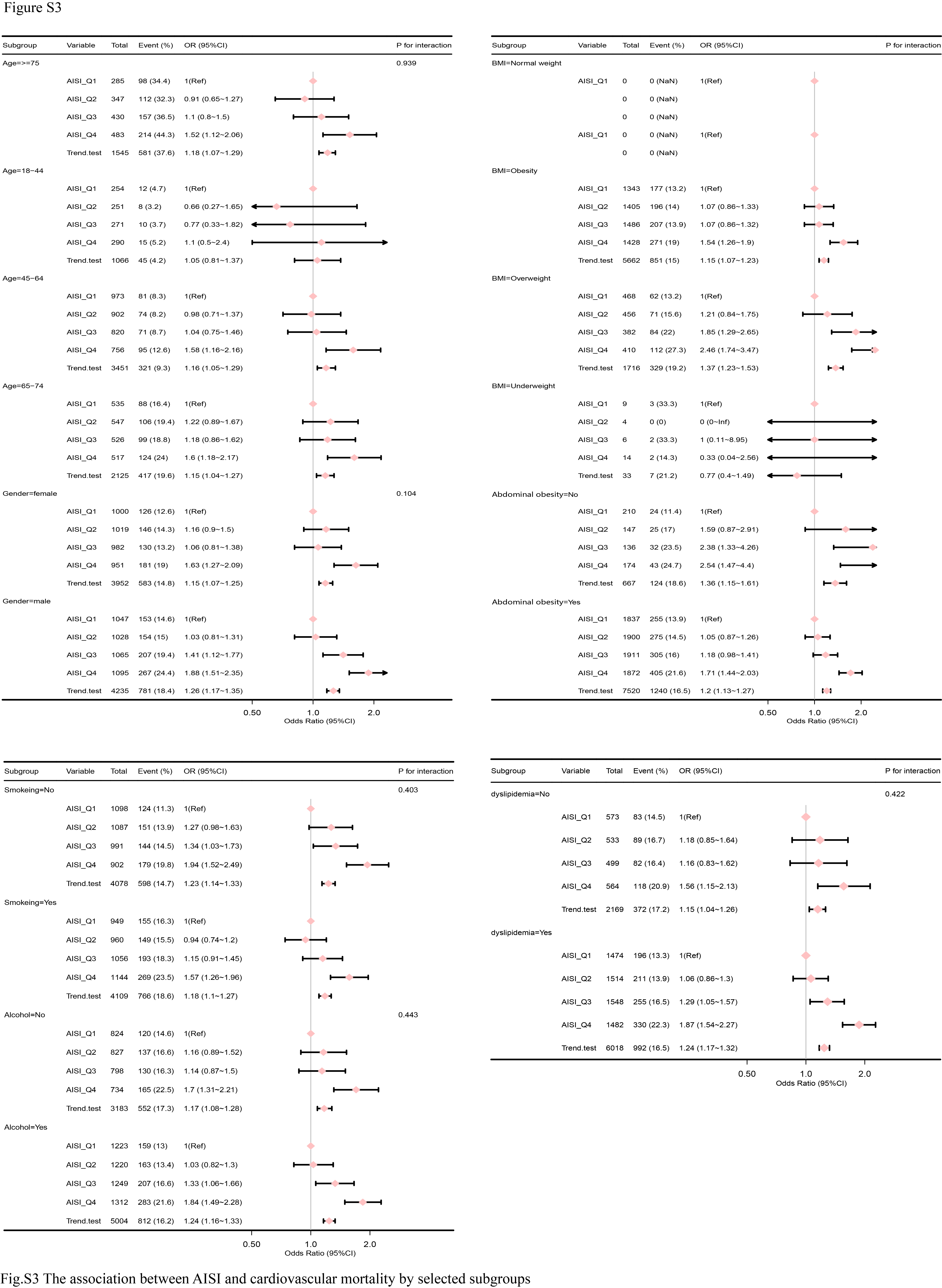

Supplement: Supplementary file 10 [file medi-105-e49713-s010.tiff]

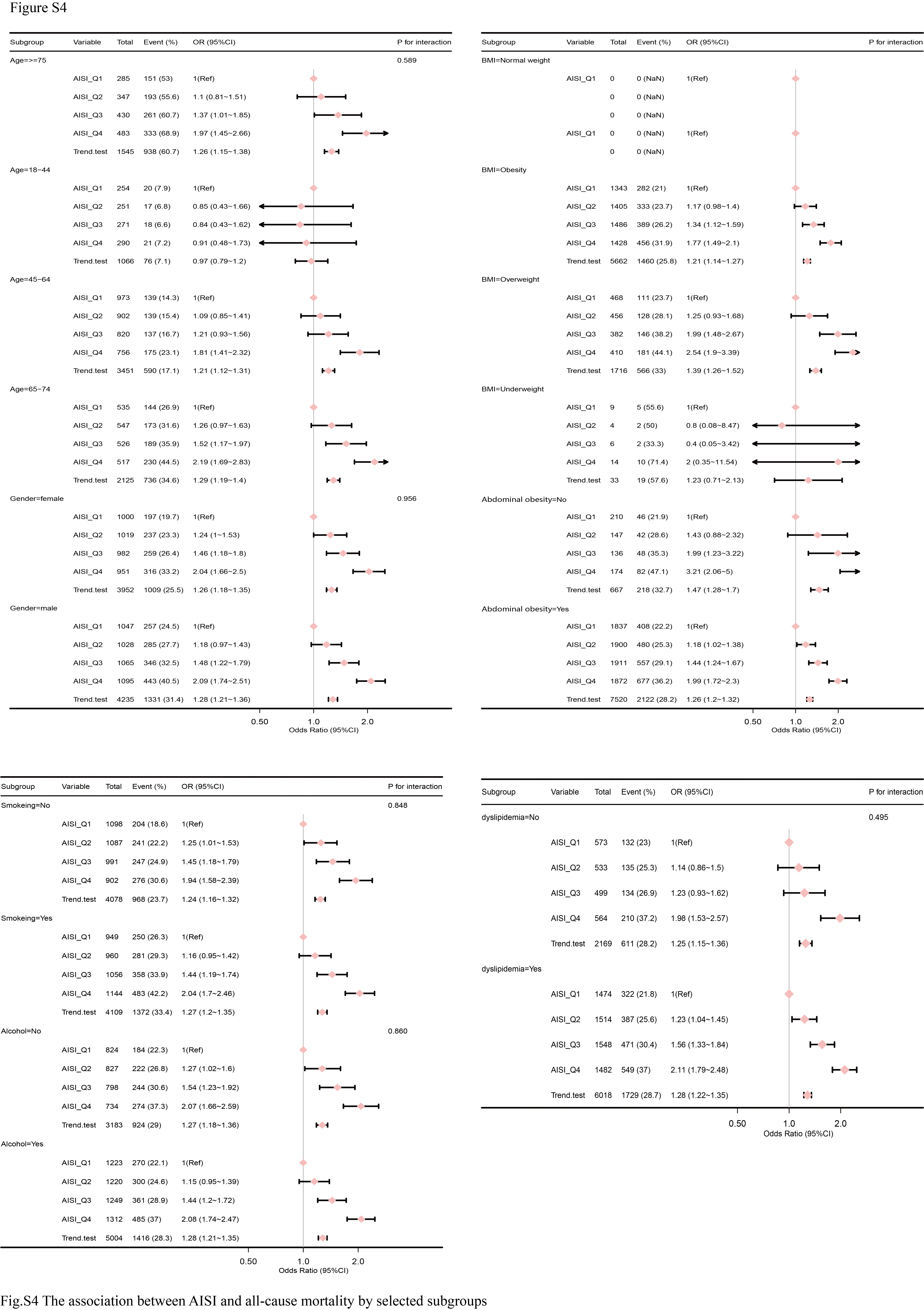

Supplement: Supplementary file 11 [file medi-105-e49713-s011.tiff]
