# Supplementary material for: Association of AISI and SIRI levels with mortality risk in patients with type 2 diabetes: A retrospective cohort study
Source: Medicine (Baltimore). 2026 Jul 17;105(29):e49713. doi: 10.1097/MD.0000000000049713 (PMC13384559; doi:10.1097/MD.0000000000049713)
Supplement: Supplementary file 2 [file medi-105-e49713-s002.docx]

TABLE S1 Baseline characteristics of patients stratified by SIRI levels.

| **Characteristic** | **Overall**  N = 8,187 | **Q1**  N = 2,047 | **Q2**  N = 2,047 | **Q3**  N = 2,047 | **Q4**  N = 2,046 | **p-value** |
| --- | --- | --- | --- | --- | --- | --- |
| **Demographic characteristics** | | | | | | |
| **Age**, n (%) |  |  |  |  |  | < 0.001 |
| 18-44 | 1066 (13.0) | 254 (12.4) | 251 (12.3) | 271 (13.2) | 290 (14.2) |  |
| 45-64 | 3451 (42.2) | 973 (47.5) | 902 (44.1) | 820 (40.1) | 756 (37) |  |
| 65-74 | 2125 (26.0) | 535 (26.1) | 547 (26.7) | 526 (25.7) | 517 (25.3) |  |
| >=75 | 1545 (18.9) | 285 (13.9) | 347 (17) | 430 (21) | 483 (23.6) |  |
| **Gender**, n (%) |  |  |  |  |  | 0.184 |
| female | 3952 (48.3) | 1000 (48.9) | 1019 (49.8) | 982 (48) | 951 (46.5) |  |
| male | 4235 (51.7) | 1047 (51.1) | 1028 (50.2) | 1065 (52) | 1095 (53.5) |  |
| **Race**, n (%) |  |  |  |  |  | < 0.001 |
| Mexican American | 1679 (20.5) | 394 (19.2) | 491 (24) | 434 (21.2) | 360 (17.6) |  |
| Non-Hispanic Black | 2056 (25.1) | 767 (37.5) | 531 (25.9) | 410 (20) | 348 (17) |  |
| Non-Hispanic White | 2932 (35.8) | 472 (23.1) | 645 (31.5) | 818 (40) | 997 (48.7) |  |
| Other | 1520 (18.6) | 414 (20.2) | 380 (18.6) | 385 (18.8) | 341 (16.7) |  |
| **Marital**, n(%) |  |  |  |  |  | 0.209 |
| No | 3360 (41.0) | 815 (39.8) | 839 (41) | 828 (40.4) | 878 (42.9) |  |
| Yes | 4827 (59.0) | 1232 (60.2) | 1208 (59) | 1219 (59.6) | 1168 (57.1) |  |
| **Education**, n (%) |  |  |  |  |  | 0.001 |
| Below high school | 3053 (37.3) | 797 (38.9) | 810 (39.6) | 744 (36.3) | 702 (34.3) |  |
| High School or above | 5134 (62.7) | 1250 (61.1) | 1237 (60.4) | 1303 (63.7) | 1344 (65.7) |  |
| **PIR**, n(%) |  |  |  |  |  | 0.779 |
| Not poor | 6441 (78.7) | 1604 (78.4) | 1598 (78.1) | 1620 (79.1) | 1619 (79.1) |  |
| Poor | 1746 (21.3) | 443 (21.6) | 449 (21.9) | 427 (20.9) | 427 (20.9) |  |
| **BMI**, n (%) |  |  |  |  |  | < 0.001 |
| Normal weight | 776 (9.5) | 227 (11.1) | 182 (8.9) | 173 (8.5) | 194 (9.5) |  |
| Obesity | 5662 (69.2) | 1343 (65.6) | 1405 (68.6) | 1486 (72.6) | 1428 (69.8) |  |
| Overweight | 1716 (21.0) | 468 (22.9) | 456 (22.3) | 382 (18.7) | 410 (20) |  |
| Underweight | 33 (0.4) | 9 (0.4) | 4 (0.2) | 6 (0.3) | 14 (0.7) |  |
| **Abdominal obesity**, n (%) |  |  |  |  |  | < 0.001 |
| No | 667 ( 8.1) | 210 (10.3) | 147 (7.2) | 136 (6.6) | 174 (8.5) |  |
| Yes | 7520 (91.9) | 1837 (89.7) | 1900 (92.8) | 1911 (93.4) | 1872 (91.5) |  |
| **Smoking**, n (%) |  |  |  |  |  | < 0.001 |
| No | 4078 (49.8) | 1098 (53.6) | 1087 (53.1) | 991 (48.4) | 902 (44.1) |  |
| Yes | 4109 (50.2) | 949 (46.4) | 960 (46.9) | 1056 (51.6) | 1144 (55.9) |  |
| **Alcohol**, n (%) |  |  |  |  |  | 0.01 |
| No | 3183 (38.9) | 824 (40.3) | 827 (40.4) | 798 (39) | 734 (35.9) |  |
| Yes | 5004 (61.1) | 1223 (59.7) | 1220 (59.6) | 1249 (61) | 1312 (64.1) |  |
| **Medical history** |  |  |  |  |  |  |
| **dyslipidemia**, n (%) |  |  |  |  |  | 0.037 |
| No | 2169 (26.5) | 573 (28) | 533 (26) | 499 (24.4) | 564 (27.6) |  |
| Yes | 6018 (73.5) | 1474 (72) | 1514 (74) | 1548 (75.6) | 1482 (72.4) |  |

Values are, n (%) or mean ± SD.

PIR, Poverty Income Ratio; BMI, body mass index.
