# Supplementary material for: Association of AISI and SIRI levels with mortality risk in patients with type 2 diabetes: A retrospective cohort study
Source: Medicine (Baltimore). 2026 Jul 17;105(29):e49713. doi: 10.1097/MD.0000000000049713 (PMC13384559; doi:10.1097/MD.0000000000049713)
Supplement: Supplementary file 3 [file medi-105-e49713-s003.docx]

TABLE S2 Baseline characteristics of patients stratified by AISI levels.

| **Characteristic** | **Overall**  N = 8,187 | **Q1**  N = 2,047 | **Q2**  N = 2,047 | **Q3**  N = 2,047 | **Q4**  N = 2,046 | **p-value** |
| --- | --- | --- | --- | --- | --- | --- |
| **Demographic characteristics** | | | | | | |
| **Age**, n (%) |  |  |  |  |  | < 0.001 |
| 18-44 | 1066 (13.0) | 303 (14.8) | 300 (14.7) | 254 (12.4) | 209 (10.2) |  |
| 45-64 | 3451 (42.2) | 1027 (50.2) | 911 (44.5) | 834 (40.7) | 679 (33.2) |  |
| 65-74 | 2125 (26.0) | 492 (24) | 543 (26.5) | 526 (25.7) | 564 (27.6) |  |
| >=75 | 1545 (18.9) | 225 (11) | 293 (14.3) | 433 (21.2) | 594 (29) |  |
| **Gender**, n (%) |  |  |  |  |  | < 0.001 |
| female | 3952 (48.3) | 1175 (57.4) | 1090 (53.2) | 897 (43.8) | 790 (38.6) |  |
| male | 4235 (51.7) | 872 (42.6) | 957 (46.8) | 1150 (56.2) | 1256 (61.4) |  |
| **Race**, n (%) |  |  |  |  |  | < 0.001 |
| Mexican American | 1679 (20.5) | 411 (20.1) | 486 (23.7) | 452 (22.1) | 330 (16.1) |  |
| Non-Hispanic Black | 2056 (25.1) | 846 (41.3) | 505 (24.7) | 391 (19.1) | 314 (15.3) |  |
| Non-Hispanic White | 2932 (35.8) | 389 (19) | 649 (31.7) | 818 (40) | 1076 (52.6) |  |
| Other | 1520 (18.6) | 401 (19.6) | 407 (19.9) | 386 (18.9) | 326 (15.9) |  |
| **Marital**, n(%) |  |  |  |  |  | 0.371 |
| No | 3360 (41.0) | 859 (42) | 848 (41.4) | 807 (39.4) | 846 (41.3) |  |
| Yes | 4827 (59.0) | 1188 (58) | 1199 (58.6) | 1240 (60.6) | 1200 (58.7) |  |
| **Education**, n (%) |  |  |  |  |  | 0.003 |
| Below high school | 3053 (37.3) | 815 (39.8) | 785 (38.3) | 748 (36.5) | 705 (34.5) |  |
| High School or above | 5134 (62.7) | 1232 (60.2) | 1262 (61.7) | 1299 (63.5) | 1341 (65.5) |  |
| **PIR**, n(%) |  |  |  |  |  | 0.107 |
| Not poor | 6441 (78.7) | 1575 (76.9) | 1624 (79.3) | 1607 (78.5) | 1635 (79.9) |  |
| Poor | 1746 (21.3) | 472 (23.1) | 423 (20.7) | 440 (21.5) | 411 (20.1) |  |
| **BMI**, n (%) |  |  |  |  |  | 0.047 |
| Normal weight | 776 (9.5) | 224 (10.9) | 177 (8.6) | 180 (8.8) | 195 (9.5) |  |
| Obesity | 5662 (69.2) | 1358 (66.3) | 1450 (70.8) | 1447 (70.7) | 1407 (68.8) |  |
| Overweight | 1716 (21.0) | 456 (22.3) | 415 (20.3) | 413 (20.2) | 432 (21.1) |  |
| Underweight | 33 (0.4) | 9 (0.4) | 5 (0.2) | 7 (0.3) | 12 (0.6) |  |
| **Abdominal obesity**, n (%) |  |  |  |  |  | < 0.001 |
| No | 667 ( 8.1) | 215 (10.5) | 151 (7.4) | 147 (7.2) | 154 (7.5) |  |
| Yes | 7520 (91.9) | 1832 (89.5) | 1896 (92.6) | 1900 (92.8) | 1892 (92.5) |  |
| **Smoking**, n (%) |  |  |  |  |  | < 0.001 |
| No | 4078 (49.8) | 1149 (56.1) | 1108 (54.1) | 946 (46.2) | 875 (42.8) |  |
| Yes | 4109 (50.2) | 898 (43.9) | 939 (45.9) | 1101 (53.8) | 1171 (57.2) |  |
| **Alcohol**, n (%) |  |  |  |  |  | < 0.001 |
| No | 3183 (38.9) | 894 (43.7) | 824 (40.3) | 780 (38.1) | 685 (33.5) |  |
| Yes | 5004 (61.1) | 1153 (56.3) | 1223 (59.7) | 1267 (61.9) | 1361 (66.5) |  |
| **Medical history** |  |  |  |  |  |  |
| **dyslipidemia**, n (%) |  |  |  |  |  | 0.008 |
| No | 2169 (26.5) | 532 (26) | 527 (25.7) | 510 (24.9) | 600 (29.3) |  |
| Yes | 6018 (73.5) | 1515 (74) | 1520 (74.3) | 1537 (75.1) | 1446 (70.7) |  |

Values are, n (%) or mean ± SD.

PIR, Poverty Income Ratio; BMI, body mass index
