# Supplementary material for: Association of AISI and SIRI levels with mortality risk in patients with type 2 diabetes: A retrospective cohort study
Source: Medicine (Baltimore). 2026 Jul 17;105(29):e49713. doi: 10.1097/MD.0000000000049713 (PMC13384559; doi:10.1097/MD.0000000000049713)
Supplement: Supplementary file 4 [file medi-105-e49713-s004.docx]

Table S3 Weighted Cox regression analysis of AISI with all-cause mortality in adults with T2DM

|  | Model 1 | | | Model 2 | | | Model 3 | | | |
| --- | --- | --- | --- | --- | --- | --- | --- | --- | --- | --- |
|  | HR | 95%CI | P-value | HR | 95%CI | P-value | HR | 95%CI | P-value | |
| All-cause mortality | | | | | | | | | | |
| Group 1 | ref | | | ref | | | ref | | | |
| Group 2 | 1.13 | (1.00–1.27) | 0.058 | 1.06 | (0.94–1.20) | 0.332 | 1.08 | (0.91–1.27) | | 0.376 |
| Group 3 | 1.32 | (1.17–1.49) | <0.001 | 1.16 | (1.02–1.30) | 0.019 | 1.17 | (0.99–1.37) | | 0.060 |
| Group 4 | 1.87 | (1.67–2.09) | <0.001 | 1.68 | (1.50–1.88) | <0.001 | 1.71 | (1.47–2.00) | | <0.001 |

Model 1: Not adjusted.

Model 2: Adjusted by age, gender.

Model 3.Adjusted by age, gender, race, education, PIR, smoking, drinking, BMI, abdominal obesity and lipid status.
