# Supplementary material for: Association of AISI and SIRI levels with mortality risk in patients with type 2 diabetes: A retrospective cohort study
Source: Medicine (Baltimore). 2026 Jul 17;105(29):e49713. doi: 10.1097/MD.0000000000049713 (PMC13384559; doi:10.1097/MD.0000000000049713)
Supplement: Supplementary file 5 [file medi-105-e49713-s005.docx]

Table S4 Weighted Cox regression analysis of SIRI with all-cause mortality in adults with T2DM

|  | Model 1 | | | Model 2 | | | Model 3 | | | |
| --- | --- | --- | --- | --- | --- | --- | --- | --- | --- | --- |
|  | HR | 95%CI | P-value | HR | 95%CI | P-value | HR | 95%CI | P-value | |
| All-cause mortality | | | | | | | | | | |
| Group 1 | ref | | | ref | | | ref | | | |
| Group 2 | 1.33 | (1.17–1.51) | < 0.001 | 1.22 | (1.07–1.38) | 0.003 | 1.26 | (1.06–1.49) | | 0.008 |
| Group 3 | 1.62 | (1.43–1.84) | < 0.001 | 1.30 | (1.15–1.48) | < 0.001 | 1.39 | (1.17–1.64) | | < 0.001 |
| Group 4 | 2.86 | (2.55–3.22) | < 0.001 | 2.05 | (1.82–2.31) | < 0.001 | 2.13 | (1.81–2.51) | | < 0.001 |

Model 1: Not adjusted.

Model 2: Adjusted by age, gender.

Model 3.Adjusted by age, gender, race, education, PIR, smoking, drinking, BMI, abdominal obesity and lipid status.
