# Supplementary material for: Association of AISI and SIRI levels with mortality risk in patients with type 2 diabetes: A retrospective cohort study
Source: Medicine (Baltimore). 2026 Jul 17;105(29):e49713. doi: 10.1097/MD.0000000000049713 (PMC13384559; doi:10.1097/MD.0000000000049713)
Supplement: Supplementary file 6 [file medi-105-e49713-s006.docx]

Table S5 Weighted Cox regression analysis of AISI with CVD mortality in adults with T2DM

|  | Model 1 | | | Model 2 | | | Model 3 | | | |
| --- | --- | --- | --- | --- | --- | --- | --- | --- | --- | --- |
|  | HR | 95%CI | P-value | HR | 95%CI | P-value | HR | 95%CI | P-value | |
| Cardiovascular mortality | | | | | | | | | | |
| Group 1 | ref | | | ref | | | ref | | | |
| Group 2 | 1.07 | (0.85–1.34) | 0.564 | 1.00 | (0.80–1.26) | 0.976 | 0.93 | (0.69–1.26) | | 0.633 |
| Group 3 | 1.18 | (0.95–1.48) | 0.142 | 1.01 | (0.80–1.26) | 0.954 | 1.00 | (0.74–1.35) | | 0.995 |
| Group 4 | 1.83 | (1.49–2.25) | <0.001 | 1.60 | (1.30–1.97) | <0.001 | 1.46 | (1.10–1.95) | | 0.009 |

Model 1: Not adjusted.

Model 2: Adjusted by age, gender.

Model 3.Adjusted by age, gender, race, education, PIR, smoking, drinking, BMI, abdominal obesity and lipid status.
