# Supplementary material for: Association of AISI and SIRI levels with mortality risk in patients with type 2 diabetes: A retrospective cohort study
Source: Medicine (Baltimore). 2026 Jul 17;105(29):e49713. doi: 10.1097/MD.0000000000049713 (PMC13384559; doi:10.1097/MD.0000000000049713)
Supplement: Supplementary file 7 [file medi-105-e49713-s007.docx]

Table S6 Weighted Cox regression analysis of SIRI with CVD mortality in adults with T2DM

|  | Model 1 | | | Model 2 | | | Model 3 | | | |
| --- | --- | --- | --- | --- | --- | --- | --- | --- | --- | --- |
|  | HR | 95%CI | P-value | HR | 95%CI | P-value | HR | 95%CI | P-value | |
| Cardiovascular mortality | | | | | | | | | | |
| Group 1 | ref | | | ref | | | ref | | | |
| Group 2 | 1.27 | (1.00–1.61) | 0.053 | 1.14 | (0.94–1.45) | 0.270 | 1.16 | (0.84–1.59) | | 0.371 |
| Group 3 | 1.45 | (1.14–1.83) | 0.002 | 1.12 | (0.88–1.42) | 0.348 | 1.18 | (0.86–1.62) | | 0.311 |
| Group 4 | 2.83 | (2.40–3.68) | < 0.001 | 2.01 | (1.61–2.50) | < 0.001 | 1.98 | (1.46–2.69) | | < 0.001 |

Model 1: Not adjusted.

Model 2: Adjusted by age, gender.

Model 3.Adjusted by age, gender, race, education, PIR, smoking, drinking, BMI, abdominal obesity and lipid status,.
