# Supplementary material for: Association of AISI and SIRI levels with mortality risk in patients with type 2 diabetes: A retrospective cohort study
Source: Medicine (Baltimore). 2026 Jul 17;105(29):e49713. doi: 10.1097/MD.0000000000049713 (PMC13384559; doi:10.1097/MD.0000000000049713)
Supplement: Supplementary file 8 [file medi-105-e49713-s008.doc]

**Table S7. Proportional hazards assumption and multicollinearity diagnostics for fully adjusted Cox models**

| Outcome | Inflammatory index | Schoenfeld residuals: index P value | Schoenfeld residuals: global P value | Maximum adjusted GVIF |
| --- | --- | --- | --- | --- |
| All-cause mortality | SIRI | 0.076 | 0.187 | 1.296 |
| Cardiovascular mortality | SIRI | 0.015 | 0.383 | 1.303 |
| All-cause mortality | AISI | 0.118 | 0.156 | 1.292 |
| Cardiovascular mortality | AISI | 0.022 | 0.468 | 1.301 |

**Abbreviations:** AISI, Aggregate Index of Systemic Inflammation; SIRI, Systemic Inflammation Response Index; PH, proportional hazards; GVIF, generalized variance inflation factor.

**Note:** The proportional hazards assumption was assessed using Schoenfeld residuals. Multicollinearity was evaluated using adjusted GVIF values, calculated as GVIF^(1/(2×Df)) for variables with multiple degrees of freedom. All maximum adjusted GVIF values were below 2, indicating no substantial multicollinearity in the fully adjusted models. For cardiovascular mortality models, the global Schoenfeld tests were not significant, but the main exposure terms showed potential non-proportionality; therefore, the corresponding HRs should be interpreted as average associations over the follow-up period.
